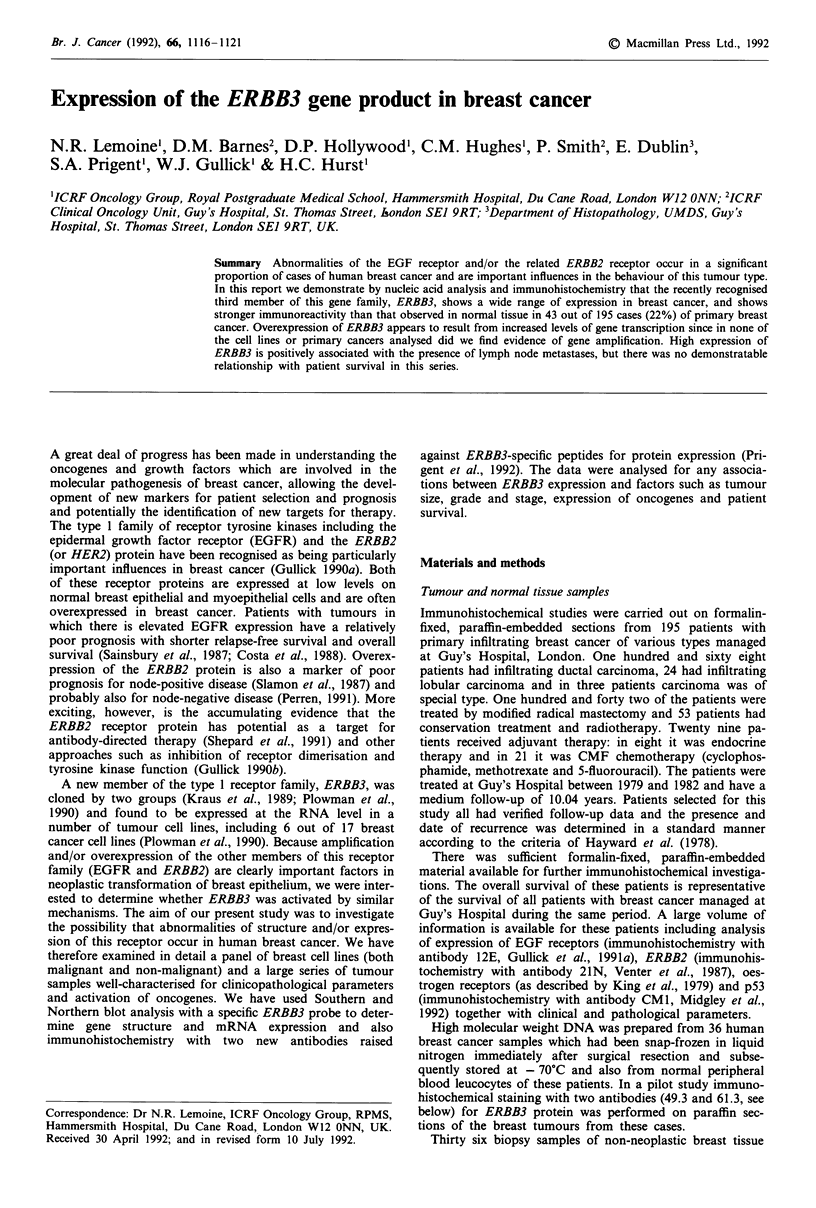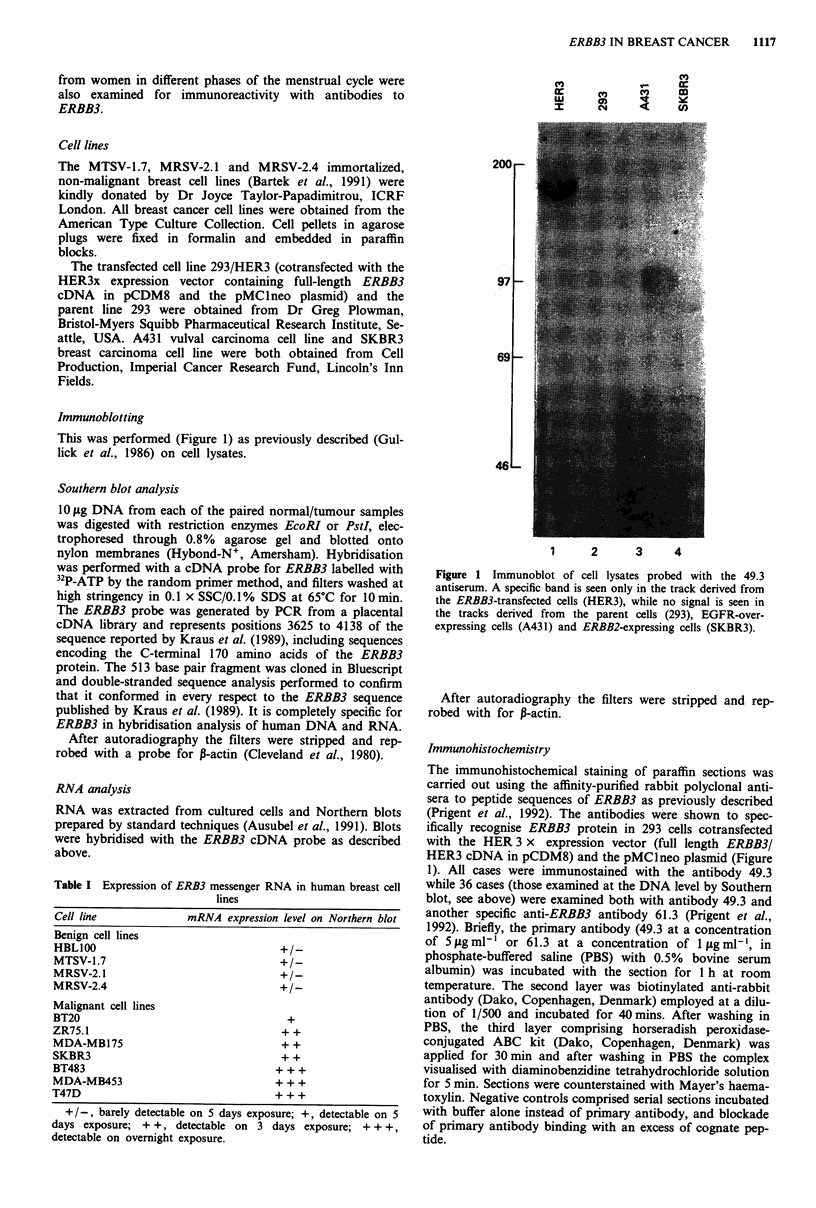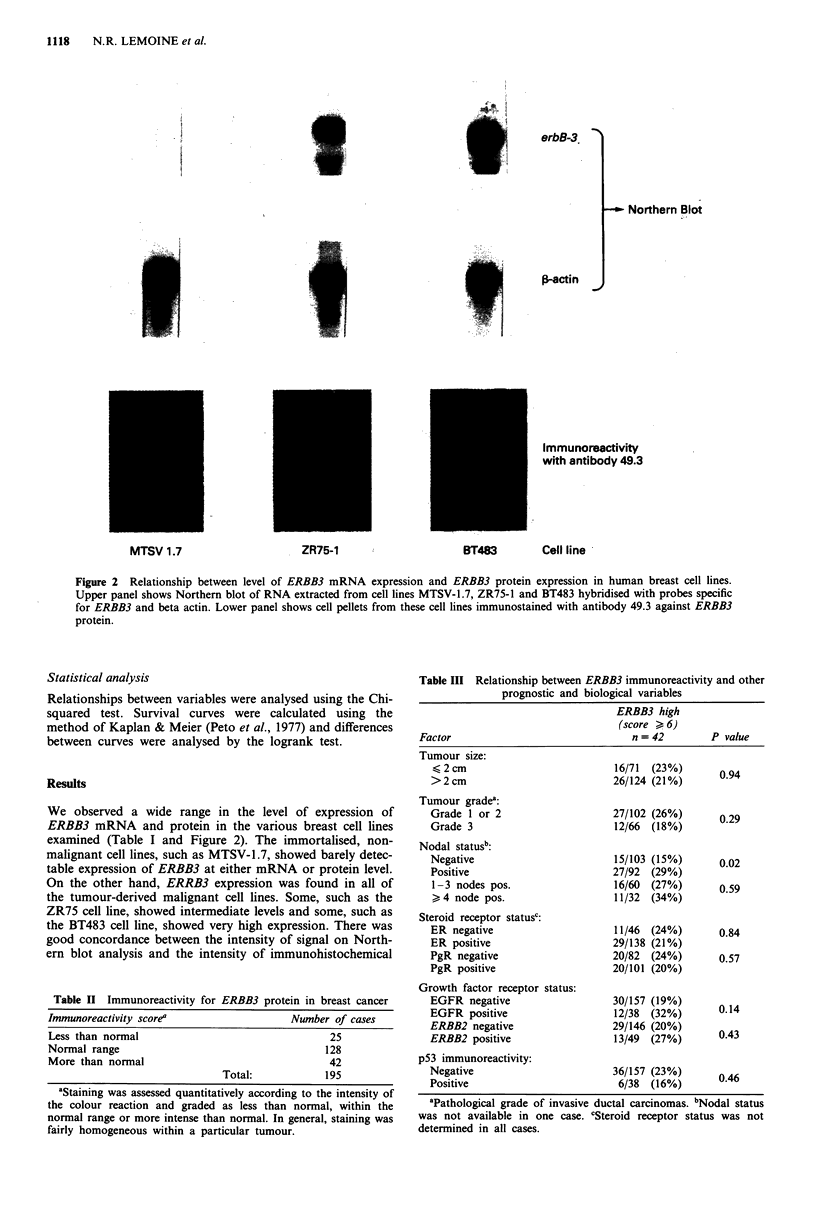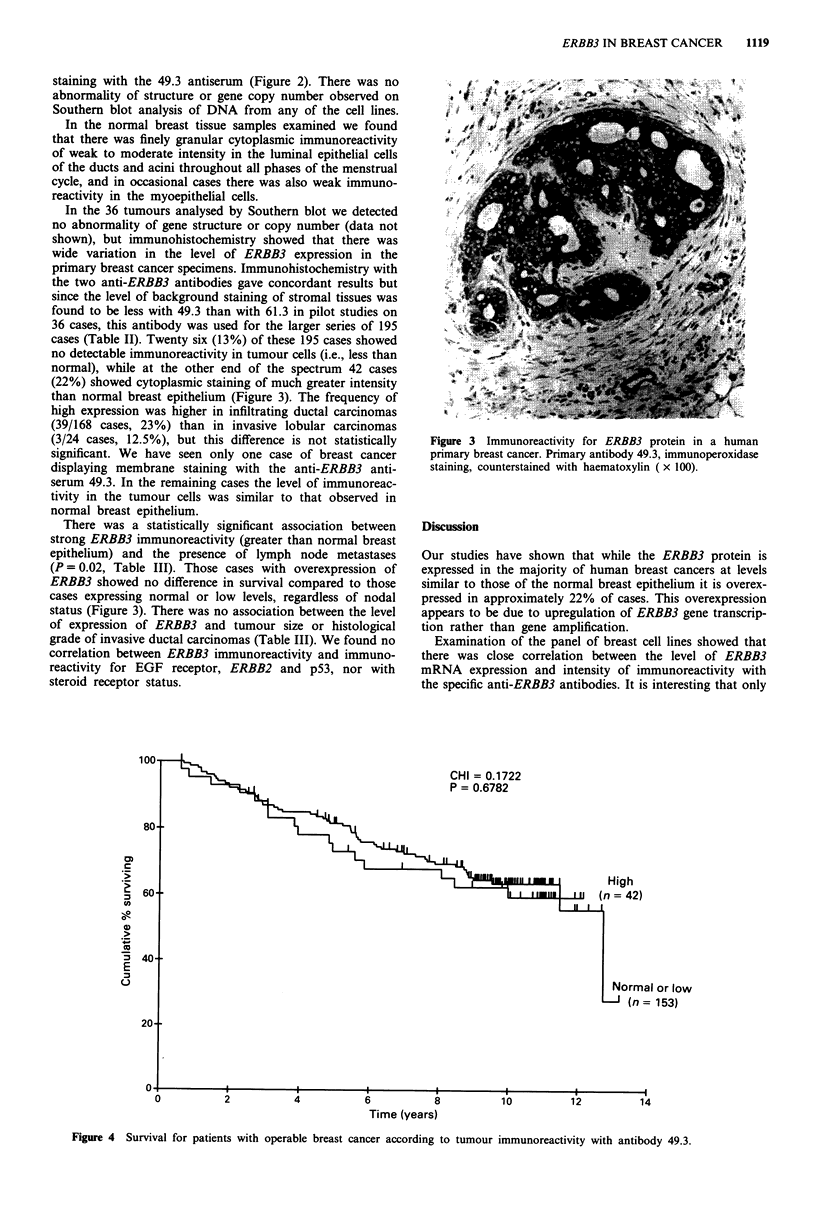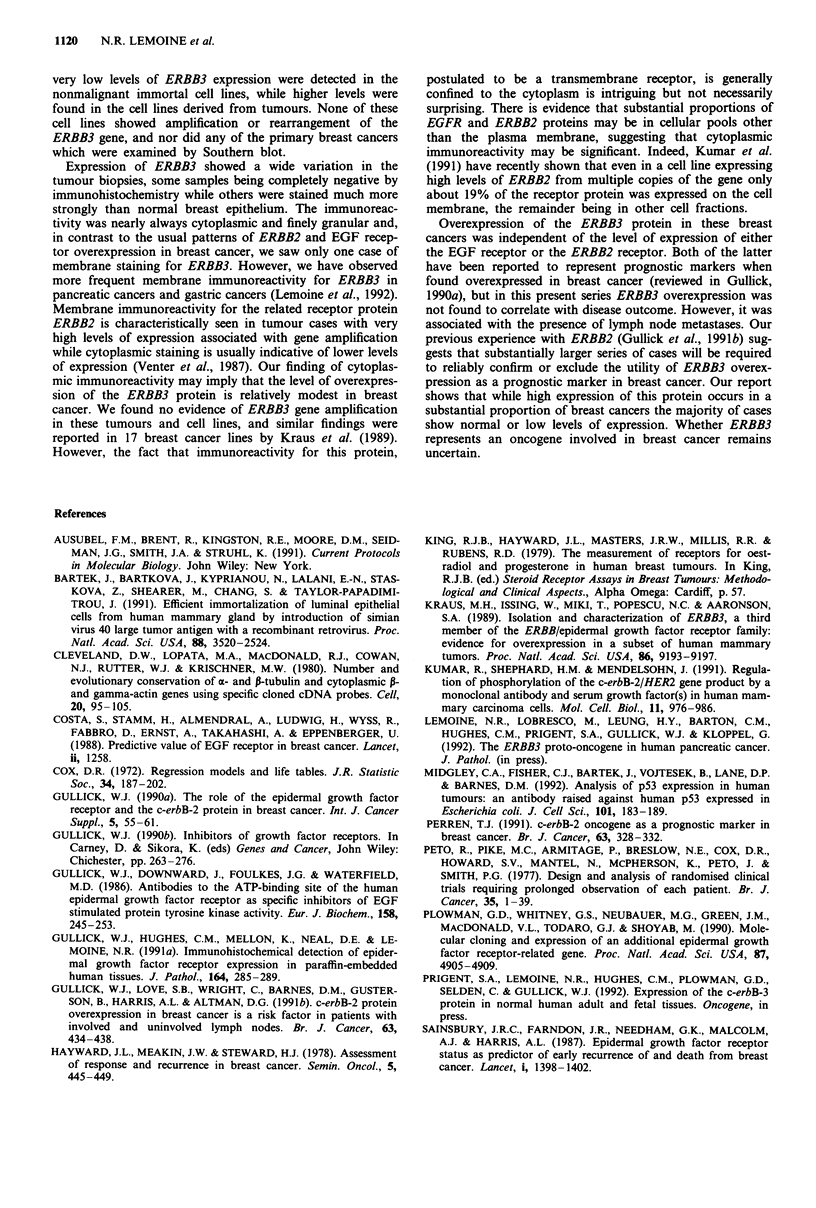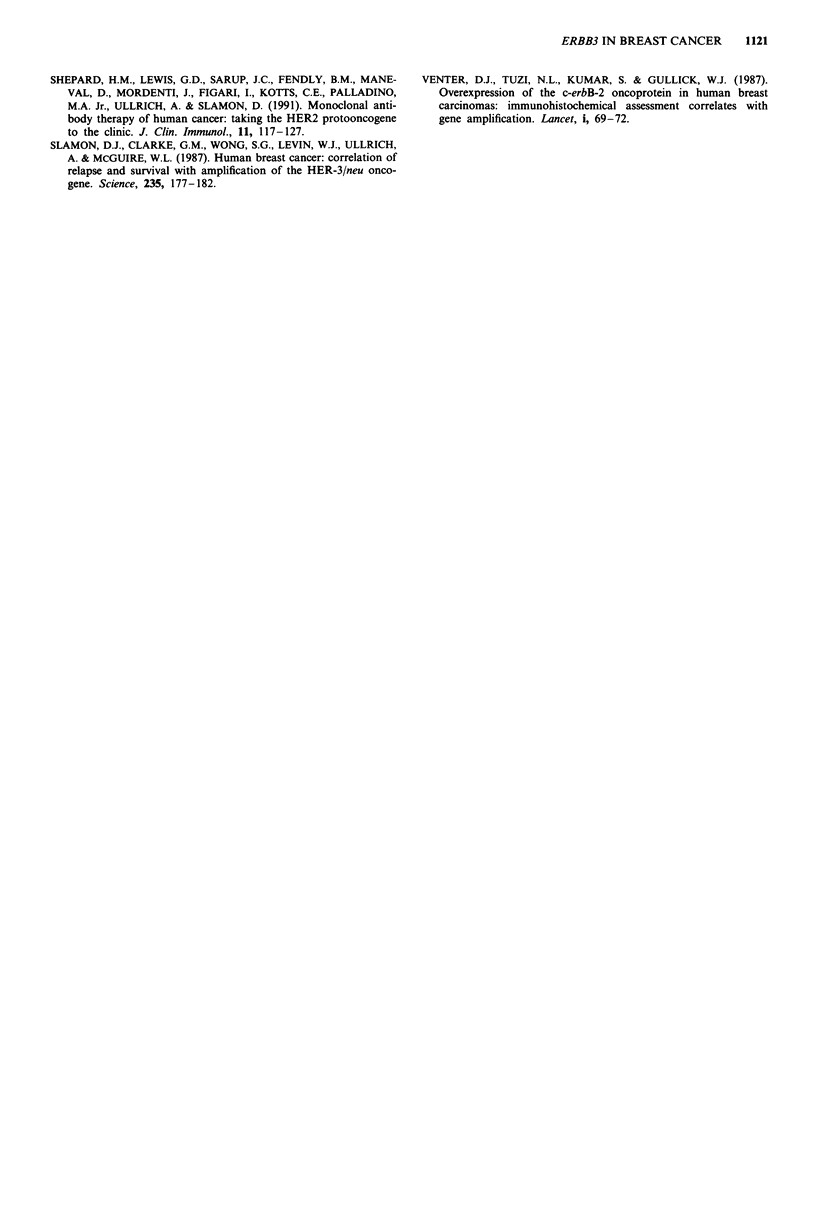# Expression of the ERBB3 gene product in breast cancer.

**DOI:** 10.1038/bjc.1992.420

**Published:** 1992-12

**Authors:** N. R. Lemoine, D. M. Barnes, D. P. Hollywood, C. M. Hughes, P. Smith, E. Dublin, S. A. Prigent, W. J. Gullick, H. C. Hurst

**Affiliations:** ICRF Oncology Group, Royal Postgraduate Medical School, Hammersmith Hospital, London, UK.

## Abstract

**Images:**


*Br. J. Cancer* (1992), **66**, 1116–1121 © Macmillan Press Ltd., 1992

### Expression of the *ERBB3* gene product in breast cancer

N.R. Lemoine[1], D.M. Barnes[2], D.P. Hollywood[1], C.M. Hughes[1], P. Smith[2], E. Dublin[3], S.A. Prigent[1], W.J. Gullick[1] & H.C. Hurst[1]

[1]*ICRF Oncology Group, Royal Postgraduate Medical School, Hammersmith Hospital, Du Cane Road, London W12 0NN;* [2]*ICRF Clinical Oncology Unit, Guy's Hospital, St. Thomas Street, London SE1 9RT;* [3]*Department of Histopathology, UMDS, Guy's Hospital, St. Thomas Street, London SE1 9RT, UK.*

**Summary** Abnormalities of the EGF receptor and/or the related *ERBB2* receptor occur in a significant proportion of cases of human breast cancer and are important influences in the behaviour of this tumour type. In this report we demonstrate by nucleic acid analysis and immunohistochemistry that the recently recognised third member of this gene family, *ERBB3*, shows a wide range of expression in breast cancer, and shows stronger immunoreactivity than that observed in normal tissue in 43 out of 195 cases (22%) of primary breast cancer. Overexpression of *ERBB3* appears to result from increased levels of gene transcription since in none of the cell lines or primary cancers analysed did we find evidence of gene amplification. High expression of *ERBB3* is positively associated with the presence of lymph node metastases, but there was no demonstratable relationship with patient survival in this series.

A great deal of progress has been made in understanding the oncogenes and growth factors which are involved in the molecular pathogenesis of breast cancer, allowing the development of new markers for patient selection and prognosis and potentially the identification of new targets for therapy. The type 1 family of receptor tyrosine kinases including the epidermal growth factor receptor (EGFR) and the *ERBB2* (or *HER2*) protein have been recognised as being particularly important influences in breast cancer (Gullick 1990*a*). Both of these receptor proteins are expressed at low levels on normal breast epithelial and myoepithelial cells and are often overexpressed in breast cancer. Patients with tumours in which there is elevated EGFR expression have a relatively poor prognosis with shorter relapse-free survival and overall survival (Sainsbury *et al.*, 1987; Costa *et al.*, 1988). Overexpression of the *ERBB2* protein is also a marker of poor prognosis for node-positive disease (Slamon *et al.*, 1987) and probably also for node-negative disease (Perren, 1991). More exciting, however, is the accumulating evidence that the *ERBB2* receptor protein has potential as a target for antibody-directed therapy (Shepard *et al.*, 1991) and other approaches such as inhibition of receptor dimerisation and tyrosine kinase function (Gullick 1990*b*).

A new member of the type 1 receptor family, *ERBB3*, was cloned by two groups (Kraus *et al.*, 1989; Plowman *et al.*, 1990) and found to be expressed at the RNA level in a number of tumour cell lines, including 6 out of 17 breast cancer cell lines (Plowman *et al.*, 1990). Because amplification and/or overexpression of the other members of this receptor family (EGFR and *ERBB2*) are clearly important factors in neoplastic transformation of breast epithelium, we were interested to determine whether *ERBB3* was activated by similar mechanisms. The aim of our present study was to investigate the possibility that abnormalities of structure and/or expression of this receptor occur in human breast cancer. We have therefore examined in detail a panel of breast cell lines (both malignant and non-malignant) and a large series of tumour samples well-characterised for clinicopathological parameters and activation of oncogenes. We have used Southern and Northern blot analysis with a specific *ERBB3* probe to determine gene structure and mRNA expression and also immunohistochemistry with two new antibodies raised against *ERBB3*-specific peptides for protein expression (Prigent *et al.*, 1992). The data were analysed for any associations between *ERBB3* expression and factors such as tumour size, grade and stage, expression of oncogenes and patient survival.

### Materials and methods

### *Tumour and normal tissue samples*

Immunohistochemical studies were carried out on formalin-fixed, paraffin-embedded sections from 195 patients with primary infiltrating breast cancer of various types managed at Guy's Hospital, London. One hundred and sixty eight patients had infiltrating ductal carcinoma, 24 had infiltrating lobular carcinoma and in three patients carcinoma was of special type. One hundred and forty two of the patients were treated by modified radical mastectomy and 53 patients had conservation treatment and radiotherapy. Twenty nine patients received adjuvant therapy: in eight it was endocrine therapy and in 21 it was CMF chemotherapy (cyclophosphamide, methotrexate and 5-fluorouracil). The patients were treated at Guy's Hospital between 1979 and 1982 and have a medium follow-up of 10.04 years. Patients selected for this study all had verified follow-up data and the presence and date of recurrence was determined in a standard manner according to the criteria of Hayward *et al.* (1978).

There was sufficient formalin-fixed, paraffin-embedded material available for further immunohistochemical investigations. The overall survival of these patients is representative of the survival of all patients with breast cancer managed at Guy's Hospital during the same period. A large volume of information is available for these patients including analysis of expression of EGF receptors (immunohistochemistry with antibody 12E, Gullick *et al.*, 1991*a*), *ERBB2* (immunohistochemistry with antibody 21N, Venter *et al.*, 1987), oestrogen receptors (as described by King *et al.*, 1979) and p53 (immunohistochemistry with antibody CM1, Midgley *et al.*, 1992) together with clinical and pathological parameters.

High molecular weight DNA was prepared from 36 human breast cancer samples which had been snap-frozen in liquid nitrogen immediately after surgical resection and subsequently stored at − 70°C and also from normal peripheral blood leucocytes of these patients. In a pilot study immunohistochemical staining with two antibodies (49.3 and 61.3, see below) for *ERBB3* protein was performed on paraffin sections of the breast tumours from these cases.

Thirty six biopsy samples of non-neoplastic breast tissue

Correspondence: Dr N.R. Lemoine, ICRF Oncology Group, RPMS, Hammersmith Hospital, Du Cane Road, London W12 0NN, UK.
Received 30 April 1992; and in revised form 10 July 1992.

from women in different phases of the menstrual cycle were also examined for immunoreactivity with antibodies to *ERBB3*.

*Cell lines*

The MTSV-1.7, MRSV-2.1 and MRSV-2.4 immortalized, non-malignant breast cell lines (Bartek *et al.*, 1991) were kindly donated by Dr Joyce Taylor-Papadimitrou, ICRF London. All breast cancer cell lines were obtained from the American Type Culture Collection. Cell pellets in agarose plugs were fixed in formalin and embedded in paraffin blocks.

The transfected cell line 293/HER3 (cotransfected with the HER3x expression vector containing full-length *ERBB3* cDNA in pCDM8 and the pMClneo plasmid) and the parent line 293 were obtained from Dr Greg Plowman, Bristol-Myers Squibb Pharmaceutical Research Institute, Seattle, USA. A431 vulval carcinoma cell line and SKBR3 breast carcinoma cell line were both obtained from Cell Production, Imperial Cancer Research Fund, Lincoln's Inn Fields.

*Immunoblotting*

This was performed (Figure 1) as previously described (Gullick *et al.*, 1986) on cell lysates.

*Southern blot analysis*

10 μg DNA from each of the paired normal/tumour samples was digested with restriction enzymes *EcoRI* or *PstI*, electrophoresed through 0.8% agarose gel and blotted onto nylon membranes (Hybond-$N^+$, Amersham). Hybridisation was performed with a cDNA probe for *ERBB3* labelled with $^{32}P$-ATP by the random primer method, and filters washed at high stringency in 0.1 × SSC/0.1% SDS at 65°C for 10 min. The *ERBB3* probe was generated by PCR from a placental cDNA library and represents positions 3625 to 4138 of the sequence reported by Kraus *et al.* (1989), including sequences encoding the C-terminal 170 amino acids of the *ERBB3* protein. The 513 base pair fragment was cloned in Bluescript and double-stranded sequence analysis performed to confirm that it conformed in every respect to the *ERBB3* sequence published by Kraus *et al.* (1989). It is completely specific for *ERBB3* in hybridisation analysis of human DNA and RNA.

After autoradiography the filters were stripped and reprobed with a probe for β-actin (Cleveland *et al.*, 1980).

*RNA analysis*

RNA was extracted from cultured cells and Northern blots prepared by standard techniques (Ausubel *et al.*, 1991). Blots were hybridised with the *ERBB3* cDNA probe as described above.

**Table I** Expression of *ERB3* messenger RNA in human breast cell lines

| *Cell line* | *mRNA expression level on Northern blot* |
|---|---|
| Benign cell lines | |
| HBL100 | +/− |
| MTSV-1.7 | +/− |
| MRSV-2.1 | +/− |
| MRSV-2.4 | +/− |
| Malignant cell lines | |
| BT20 | + |
| ZR75.1 | + + |
| MDA-MB175 | + + |
| SKBR3 | + + |
| BT483 | + + + |
| MDA-MB453 | + + + |
| T47D | + + + |

+/−, barely detectable on 5 days exposure; +, detectable on 5 days exposure; + +, detectable on 3 days exposure; + + +, detectable on overnight exposure.

**Figure 1** Immunoblot of cell lysates probed with the 49.3 antiserum. A specific band is seen only in the track derived from the *ERBB3*-transfected cells (HER3), while no signal is seen in the tracks derived from the parent cells (293), EGFR-over-expressing cells (A431) and *ERBB2*-expressing cells (SKBR3).

After autoradiography the filters were stripped and reprobed with for β-actin.

*Immunohistochemistry*

The immunohistochemical staining of paraffin sections was carried out using the affinity-purified rabbit polyclonal antisera to peptide sequences of *ERBB3* as previously described (Prigent *et al.*, 1992). The antibodies were shown to specifically recognise *ERBB3* protein in 293 cells cotransfected with the HER 3 × expression vector (full length *ERBB3*/HER3 cDNA in pCDM8) and the pMClneo plasmid (Figure 1). All cases were immunostained with the antibody 49.3 while 36 cases (those examined at the DNA level by Southern blot, see above) were examined both with antibody 49.3 and another specific anti-*ERBB3* antibody 61.3 (Prigent *et al.*, 1992). Briefly, the primary antibody (49.3 at a concentration of 5 μg $ml^{-1}$ or 61.3 at a concentration of 1 μg $ml^{-1}$, in phosphate-buffered saline (PBS) with 0.5% bovine serum albumin) was incubated with the section for 1 h at room temperature. The second layer was biotinylated anti-rabbit antibody (Dako, Copenhagen, Denmark) employed at a dilution of 1/500 and incubated for 40 mins. After washing in PBS, the third layer comprising horseradish peroxidase-conjugated ABC kit (Dako, Copenhagen, Denmark) was applied for 30 min and after washing in PBS the complex visualised with diaminobenzidine tetrahydrochloride solution for 5 min. Sections were counterstained with Mayer's haematoxylin. Negative controls comprised serial sections incubated with buffer alone instead of primary antibody, and blockade of primary antibody binding with an excess of cognate peptide.

**Figure 2** Relationship between level of ***ERBB3*** **mRNA expression and** ***ERBB3*** **protein expression in human breast cell lines. Upper panel shows Northern blot of RNA extracted from cell lines MTSV-1.7, ZR75-1 and BT483 hybridised with probes specific for** ***ERBB3*** **and beta actin. Lower panel shows cell pellets from these cell lines immunostained with antibody 49.3 against** ***ERBB3*** **protein.**

### *Statistical analysis*

Relationships between variables were analysed using the Chi-squared test. Survival curves were calculated using the method of Kaplan & Meier (Peto *et al.*, 1977) and differences between curves were analysed by the logrank test.

### Results

We observed a wide range in the level of expression of *ERBB3* mRNA and protein in the various breast cell lines examined (Table I and Figure 2). The immortalised, non-malignant cell lines, such as MTSV-1.7, showed barely detectable expression of *ERBB3* at either mRNA or protein level. On the other hand, *ERRB3* expression was found in all of the tumour-derived malignant cell lines. Some, such as the ZR75 cell line, showed intermediate levels and some, such as the BT483 cell line, showed very high expression. There was good concordance between the intensity of signal on Northern blot analysis and the intensity of immunohistochemical

**Table II** Immunoreactivity for *ERBB3* protein in breast cancer

| *Immunoreactivity score*[a] | *Number of cases* |
|---|---|
| Less than normal | 25 |
| Normal range | 128 |
| More than normal | 42 |
| Total: | 195 |

[a]Staining was assessed quantitatively according to the intensity of the colour reaction and graded as less than normal, within the normal range or more intense than normal. In general, staining was fairly homogeneous within a particular tumour.

**Table III** Relationship between *ERBB3* immunoreactivity and other prognostic and biological variables

| *Factor* | *ERBB3 high (score ⩾ 6) n = 42* | *P value* |
|---|---|---|
| Tumour size: | | |
| ⩽ 2 cm | 16/71 (23%) | 0.94 |
| > 2 cm | 26/124 (21%) | |
| Tumour grade[a]: | | |
| Grade 1 or 2 | 27/102 (26%) | 0.29 |
| Grade 3 | 12/66 (18%) | |
| Nodal status[b]: | | |
| Negative | 15/103 (15%) | 0.02 |
| Positive | 27/92 (29%) | |
| 1-3 nodes pos. | 16/60 (27%) | 0.59 |
| ⩾ 4 node pos. | 11/32 (34%) | |
| Steroid receptor status[c]: | | |
| ER negative | 11/46 (24%) | 0.84 |
| ER positive | 29/138 (21%) | |
| PgR negative | 20/82 (24%) | 0.57 |
| PgR positive | 20/101 (20%) | |
| Growth factor receptor status: | | |
| EGFR negative | 30/157 (19%) | 0.14 |
| EGFR positive | 12/38 (32%) | |
| *ERBB2* negative | 29/146 (20%) | 0.43 |
| *ERBB2* positive | 13/49 (27%) | |
| p53 immunoreactivity: | | |
| Negative | 36/157 (23%) | 0.46 |
| Positive | 6/38 (16%) | |

[a]Pathological grade of invasive ductal carcinomas. [b]Nodal status was not available in one case. [c]Steroid receptor status was not determined in all cases.

staining with the 49.3 antiserum (Figure 2). There was no abnormality of structure or gene copy number observed on Southern blot analysis of DNA from any of the cell lines.

In the normal breast tissue samples examined we found that there was finely granular cytoplasmic immunoreactivity of weak to moderate intensity in the luminal epithelial cells of the ducts and acini throughout all phases of the menstrual cycle, and in occasional cases there was also weak immunoreactivity in the myoepithelial cells.

In the 36 tumours analysed by Southern blot we detected no abnormality of gene structure or copy number (data not shown), but immunohistochemistry showed that there was wide variation in the level of *ERBB3* expression in the primary breast cancer specimens. Immunohistochemistry with the two anti-*ERBB3* antibodies gave concordant results but since the level of background staining of stromal tissues was found to be less with 49.3 than with 61.3 in pilot studies on 36 cases, this antibody was used for the larger series of 195 cases (Table II). Twenty six (13%) of these 195 cases showed no detectable immunoreactivity in tumour cells (i.e., less than normal), while at the other end of the spectrum 42 cases (22%) showed cytoplasmic staining of much greater intensity than normal breast epithelium (Figure 3). The frequency of high expression was higher in infiltrating ductal carcinomas (39/168 cases, 23%) than in invasive lobular carcinomas (3/24 cases, 12.5%), but this difference is not statistically significant. We have seen only one case of breast cancer displaying membrane staining with the anti-*ERBB3* antiserum 49.3. In the remaining cases the level of immunoreactivity in the tumour cells was similar to that observed in normal breast epithelium.

There was a statistically significant association between strong *ERBB3* immunoreactivity (greater than normal breast epithelium) and the presence of lymph node metastases ($P = 0.02$, Table III). Those cases with overexpression of *ERBB3* showed no difference in survival compared to those cases expressing normal or low levels, regardless of nodal status (Figure 3). There was no association between the level of expression of *ERBB3* and tumour size or histological grade of invasive ductal carcinomas (Table III). We found no correlation between *ERBB3* immunoreactivity and immunoreactivity for EGF receptor, *ERBB2* and p53, nor with steroid receptor status.

**Figure 3** Immunoreactivity for *ERBB3* protein in a human primary breast cancer. Primary antibody 49.3, immunoperoxidase staining, counterstained with haematoxylin ( × 100).

### Discussion

Our studies have shown that while the *ERBB3* protein is expressed in the majority of human breast cancers at levels similar to those of the normal breast epithelium it is overexpressed in approximately 22% of cases. This overexpression appears to be due to upregulation of *ERBB3* gene transcription rather than gene amplification.

Examination of the panel of breast cell lines showed that there was close correlation between the level of *ERBB3* mRNA expression and intensity of immunoreactivity with the specific anti-*ERBB3* antibodies. It is interesting that only

**Figure 4** Survival for patients with operable breast cancer according to tumour immunoreactivity with antibody 49.3.

very low levels of *ERBB3* expression were detected in the nonmalignant immortal cell lines, while higher levels were found in the cell lines derived from tumours. None of these cell lines showed amplification or rearrangement of the *ERBB3* gene, and nor did any of the primary breast cancers which were examined by Southern blot.

Expression of *ERBB3* showed a wide variation in the tumour biopsies, some samples being completely negative by immunohistochemistry while others were stained much more strongly than normal breast epithelium. The immunoreactivity was nearly always cytoplasmic and finely granular and, in contrast to the usual patterns of *ERBB2* and EGF receptor overexpression in breast cancer, we saw only one case of membrane staining for *ERBB3*. However, we have observed more frequent membrane immunoreactivity for *ERBB3* in pancreatic cancers and gastric cancers (Lemoine *et al*., 1992). Membrane immunoreactivity for the related receptor protein *ERBB2* is characteristically seen in tumour cases with very high levels of expression associated with gene amplification while cytoplasmic staining is usually indicative of lower levels of expression (Venter *et al*., 1987). Our finding of cytoplasmic immunoreactivity may imply that the level of overexpression of the *ERBB3* protein is relatively modest in breast cancer. We found no evidence of *ERBB3* gene amplification in these tumours and cell lines, and similar findings were reported in 17 breast cancer lines by Kraus *et al*. (1989). However, the fact that immunoreactivity for this protein, postulated to be a transmembrane receptor, is generally confined to the cytoplasm is intriguing but not necessarily surprising. There is evidence that substantial proportions of *EGFR* and *ERBB2* proteins may be in cellular pools other than the plasma membrane, suggesting that cytoplasmic immunoreactivity may be significant. Indeed, Kumar *et al*. (1991) have recently shown that even in a cell line expressing high levels of *ERBB2* from multiple copies of the gene only about 19% of the receptor protein was expressed on the cell membrane, the remainder being in other cell fractions.

Overexpression of the *ERBB3* protein in these breast cancers was independent of the level of expression of either the EGF receptor or the *ERBB2* receptor. Both of the latter have been reported to represent prognostic markers when found overexpressed in breast cancer (reviewed in Gullick, 1990*a*), but in this present series *ERBB3* overexpression was not found to correlate with disease outcome. However, it was associated with the presence of lymph node metastases. Our previous experience with *ERBB2* (Gullick *et al*., 1991*b*) suggests that substantially larger series of cases will be required to reliably confirm or exclude the utility of *ERBB3* overexpression as a prognostic marker in breast cancer. Our report shows that while high expression of this protein occurs in a substantial proportion of breast cancers the majority of cases show normal or low levels of expression. Whether *ERBB3* represents an oncogene involved in breast cancer remains uncertain.

### References

AUSUBEL, F.M., BRENT, R., KINGSTON, R.E., MOORE, D.M., SEIDMAN, J.G., SMITH, J.A. & STRUHL, K. (1991). *Current Protocols in Molecular Biology*. John Wiley: New York.

BARTEK, J., BARTKOVA, J., KYPRIANOU, N., LALANI, E.-N., STASKOVA, Z., SHEARER, M., CHANG, S. & TAYLOR-PAPADIMITROU, J. (1991). Efficient immortalization of luminal epithelial cells from human mammary gland by introduction of simian virus 40 large tumor antigen with a recombinant retrovirus. *Proc. Natl. Acad. Sci. USA*, **88**, 3520-2524.

CLEVELAND, D.W., LOPATA, M.A., MACDONALD, R.J., COWAN, N.J., RUTTER, W.J. & KRISCHNER, M.W. (1980). Number and evolutionary conservation of α- and β-tubulin and cytoplasmic β- and gamma-actin genes using specific cloned cDNA probes. *Cell*, **20**, 95-105.

COSTA, S., STAMM, H., ALMENDRAL, A., LUDWIG, H., WYSS, R., FABBRO, D., ERNST, A., TAKAHASHI, A. & EPPENBERGER, U. (1988). Predictive value of EGF receptor in breast cancer. *Lancet*, **ii**, 1258.

COX, D.R. (1972). Regression models and life tables. *J.R. Statistic Soc*., **34**, 187-202.

GULLICK, W.J. (1990*a*). The role of the epidermal growth factor receptor and the c-*erb*B-2 protein in breast cancer. *Int. J. Cancer Suppl*., **5**, 55-61.

GULLICK, W.J. (1990*b*). Inhibitors of growth factor receptors. In Carney, D. & Sikora, K. (eds) *Genes and Cancer*, John Wiley: Chichester, pp. 263-276.

GULLICK, W.J., DOWNWARD, J., FOULKES, J.G. & WATERFIELD, M.D. (1986). Antibodies to the ATP-binding site of the human epidermal growth factor receptor as specific inhibitors of EGF stimulated protein tyrosine kinase activity. *Eur. J. Biochem*., **158**, 245-253.

GULLICK, W.J., HUGHES, C.M., MELLON, K., NEAL, D.E. & LEMOINE, N.R. (1991*a*). Immunohistochemical detection of epidermal growth factor receptor expression in paraffin-embedded human tissues. *J. Pathol*., **164**, 285-289.

GULLICK, W.J., LOVE, S.B., WRIGHT, C., BARNES, D.M., GUSTERSON, B., HARRIS, A.L. & ALTMAN, D.G. (1991*b*). c-*erb*B-2 protein overexpression in breast cancer is a risk factor in patients with involved and uninvolved lymph nodes. *Br. J. Cancer*, **63**, 434-438.

HAYWARD, J.L., MEAKIN, J.W. & STEWARD, H.J. (1978). Assessment of response and recurrence in breast cancer. *Semin. Oncol*., **5**, 445-449.

KING, R.J.B., HAYWARD, J.L., MASTERS, J.R.W., MILLIS, R.R. & RUBENS, R.D. (1979). The measurement of receptors for oestradiol and progesterone in human breast tumours. In King, R.J.B. (ed.) *Steroid Receptor Assays in Breast Tumours: Methodological and Clinical Aspects*., Alpha Omega: Cardiff, p. 57.

KRAUS, M.H., ISSING, W., MIKI, T., POPESCU, N.C. & AARONSON, S.A. (1989). Isolation and characterization of *ERBB3*, a third member of the *ERBB*/epidermal growth factor receptor family: evidence for overexpression in a subset of human mammary tumors. *Proc. Natl. Acad. Sci. USA*, **86**, 9193-9197.

KUMAR, R., SHEPHARD, H.M. & MENDELSOHN, J. (1991). Regulation of phosphorylation of the c-*erb*B-2/*HER*2 gene product by a monoclonal antibody and serum growth factor(s) in human mammary carcinoma cells. *Mol. Cell. Biol*., **11**, 976-986.

LEMOINE, N.R., LOBRESCO, M., LEUNG, H.Y., BARTON, C.M., HUGHES, C.M., PRIGENT, S.A., GULLICK, W.J. & KLOPPEL, G. (1992). The *ERBB3* proto-oncogene in human pancreatic cancer. *J. Pathol*. (in press).

MIDGLEY, C.A., FISHER, C.J., BARTEK, J., VOJTESEK, B., LANE, D.P. & BARNES, D.M. (1992). Analysis of p53 expression in human tumours: an antibody raised against human p53 expressed in *Escherichia coli*. *J. Cell Sci*., **101**, 183-189.

PERREN, T.J. (1991). c-*erb*B-2 oncogene as a prognostic marker in breast cancer. *Br. J. Cancer*, **63**, 328-332.

PETO, R., PIKE, M.C., ARMITAGE, P., BRESLOW, N.E., COX, D.R., HOWARD, S.V., MANTEL, N., MCPHERSON, K., PETO, J. & SMITH, P.G. (1977). Design and analysis of randomised clinical trials requiring prolonged observation of each patient. *Br. J. Cancer*, **35**, 1-39.

PLOWMAN, G.D., WHITNEY, G.S., NEUBAUER, M.G., GREEN, J.M., MACDONALD, V.L., TODARO, G.J. & SHOYAB, M. (1990). Molecular cloning and expression of an additional epidermal growth factor receptor-related gene. *Proc. Natl. Acad. Sci. USA*, **87**, 4905-4909.

PRIGENT, S.A., LEMOINE, N.R., HUGHES, C.M., PLOWMAN, G.D., SELDEN, C. & GULLICK, W.J. (1992). Expression of the c-*erb*B-3 protein in normal human adult and fetal tissues. *Oncogene*, in press.

SAINSBURY, J.R.C., FARNDON, J.R., NEEDHAM, G.K., MALCOLM, A.J. & HARRIS, A.L. (1987). Epidermal growth factor receptor status as predictor of early recurrence of and death from breast cancer. *Lancet*, **i**, 1398-1402.

SHEPARD, H.M., LEWIS, G.D., SARUP, J.C., FENDLY, B.M., MANEVAL, D., MORDENTI, J., FIGARI, I., KOTTS, C.E., PALLADINO, M.A. Jr., ULLRICH, A. & SLAMON, D. (1991). Monoclonal antibody therapy of human cancer: taking the HER2 protooncogene to the clinic. *J. Clin. Immunol.*, **11**, 117–127.

SLAMON, D.J., CLARKE, G.M., WONG, S.G., LEVIN, W.J., ULLRICH, A. & MCGUIRE, W.L. (1987). Human breast cancer: correlation of relapse and survival with amplification of the HER-3/*neu* oncogene. *Science*, **235**, 177–182.

VENTER, D.J., TUZI, N.L., KUMAR, S. & GULLICK, W.J. (1987). Overexpression of the c-*erb*B-2 oncoprotein in human breast carcinomas: immunohistochemical assessment correlates with gene amplification. *Lancet*, **i**, 69–72.